# Multimodal treatment, including extracorporeal shock wave therapy, for refractory chronic tension-type headache: a case report

**DOI:** 10.1186/s13256-023-04092-9

**Published:** 2023-10-31

**Authors:** Toru Takekawa, Toshifumi Chino, Naoki Yamada, Shu Watanabe, Masahiro Abo, Renpei Sengoku

**Affiliations:** 1 Department of Rehabilitation Medicine, School of Medicine, The Jikei University, 3-19-18, Nishi-Shimbashi, Minato-ku, Tokyo, 105-8471 Japan; 2 Department of Neurology, The Jikei University Daisan Hospital, 4-11-1, Izumihoncho, Komae-shi, Tokyo, 201-8601 Japan

**Keywords:** Case reports, Extracorporeal shock wave therapy, Medicine, Kampo, Pain, Intractable, Trigger points

## Abstract

**Background:**

Few reports have described multidisciplinary treatment, including extracorporeal shock wave therapy, for patients with refractory chronic tension-type headache. In this study, we conducted multidisciplinary treatment for a patient with chronic tension-type headache who suffered from chronic headache refractory to treatment.

**Case presentation:**

The patient was a 45-year-old Japanese male suffering from 20 years of headache. As his headache had worsened recently, he visited a local clinic. With the diagnosis of suspected tension-type headache, its treatment was unsuccessful and he was referred to our hospital. The neurology department confirmed the tension-type headache and prescribed another medication, but he showed no improvement. Then, the patient was referred to the rehabilitation medicine department for consultation. At the initial visit, we identified multiple myofascial trigger points in his bilateral posterior neck and upper back regions. At the initial visit, he was prescribed 10 mL of 1% lidocaine injected into the muscles in these areas. In addition, he received 2000 extracorporeal shock wave therapy into bilateral trapezius muscles, and was instructed to take oral *Kakkonto* extract granules, benfotiamine, pyridoxine hydrochloride, and cyanocobalamin. Cervical muscle and shoulder girdle stretches and exercises were also recommended. At follow-up treatment visits, we used extracorporeal shock wave therapy to bilateral trapezius muscles, which led to immediate pain relief. After 11 weeks, he was not taking any medication and his headache was subjectively improved and his medical treatment ended.

**Conclusion:**

A patient with chronic tension-type headache refractory to regular treatment was successfully treated with a multimodal approach including extracorporeal shock wave therapy in addition to standard treatment. For patients with tension-type headache accompanied by myofascial trigger points, it may be recommended to promptly consider aggressive multimodal treatment that includes extracorporeal shock wave therapy.

## Background

Chronic tension-type headache (CTTH), along with chronic migraine, is included in a group of headache syndromes as chronic daily headache (CDH) [1]. Treatment includes oral medications, trigger point injections, and botulinum toxin (BoNT) therapy, but treatment can be difficult [1].

The presence of pericranial tenderness is well known in TTH [2]. Typically, pain is caused by peripheral mechanisms, most likely due to increased input from myofascial nociceptors [2]. TTH is associated with significantly higher pericranial muscle tenderness, while CTTH is associated with inadequate muscle relaxation at rest [3].

Extracorporeal shock wave therapy (ESWT) (radial pressure wave, RPW) has both pain-relieving and tissue-repairing effects, and has recently been applied in orthopedic conditions such as plantar fasciitis. However, there are few reports of multidisciplinary treatment including ESWT for patients with refractory CTTH. We previously concluded that ESWT should be considered as a treatment option for TTH in the presence of TrPs [4]. This case report adds to our previous report [4] and discusses the mechanism of headache occurrence in more detail, with a particular focus on the "vicious cycle of pain.” The use of ESWT for TTH was performed after obtaining permission from the hospital director. This case report was approved by the Ethics Committee of the Jikei University School of Medicine for Biomedical Research [approval number 33-276(10,894)], and the patient gave consent for the case report.

## Case presentation

A 45-year-old Japanese male patient reported headache for 20 years prior to his visit to our hospital. His headache symptoms came in waves, but the symptoms were essentially continuous. He had a habit of drinking 1 L of beer three times a week, and he had smoked 15 cigarettes per day since the age of 20, but quit smoking at 40 years old. He had a history of bronchial asthma and allergic rhinitis. He had not used headache medication frequently, there were no particular problems with his psychosocial history, but his family history included a grandfather and an uncle who also suffered from bronchial asthma. His occupation consisted mainly of desk work. During and after the coronavirus disease 2019 (COVID-19) pandemic, he worked remotely, and at the time of his visit to our hospital, he was engaged in work on a personal computer most days.

From around March of the year that he visited our hospital, he went to a local clinic because his headache had worsened. Around this time, his symptoms of neck and shoulder heaviness worsened, likely due to stress from his child’s upcoming entrance examination and the death of his parents. In addition, he rarely went out since beginning working at home due to the COVID-19 pandemic. He felt a squeezing pain all over his head and heaviness in both of his shoulders. His headache symptoms were suspected to be TTH, and he was prescribed tiaramide hydrochloride and eperisone hydrochloride, but there was little improvement. In addition, he had severe headache symptoms that kept him in bed in the morning several times a month. He began to fall asleep when he went to events such as graduations, entrance ceremonies, and funeral services. He was referred to our neurosurgery department in May because his headaches did not improve. There were no significant findings on computed tomography (CT), and he was diagnosed with physiologic headache and placed under observation. In June the patient was further referred to our neurology department, where he was diagnosed with TTH with exclusion of intracranial organic lesions with head CT scan as well as the accompanying shoulder stiffness. He was prescribed oral eperisone hydrochloride (50 mg) one tablet three times daily. The stiffness in his shoulders improved slightly, but his headache persisted, and he was now taking loxoprofen sodium hydrate (60 mg) one tablet frequently. Because his headache and heavy-headed feeling did not improve, he was referred to our department in July for botulinum toxin type A (BoNT-A) injection (day T). At that time, the drugs he was prescribed were olopatadine hydrochloride, fluticasone propionate nasal drops, and budesonide and formoterol fumarate hydrate inhalant, which were prescribed for bronchial asthma and allergic rhinitis by a local clinic, in addition to tiaramide hydrochloride, eperisone hydrochloride, and loxoprofen sodium hydrate (60 mg) one tablet three times daily.

At the time of his initial examination, he was 171 cm tall, weighed 62 kg, and had a body mass index (BMI) of 21.2. He had no upper extremity fine motor deficits or sensory deficits. He had multiple myofascial trigger points (TrPs) in the bilateral posterior neck and upper back. Blood tests showed high γ-glutamyl transpeptidase (γ-GTP; 103 U/L), but there were no other findings of note, and the abnormal values were thought to be due to alcohol consumption. A CT scan of the head showed no obvious intracranial lesions. Before the start of treatment, his headache was rated at visual analog scale (VAS) 8.1/10, numerical rating scale (NRS) 8/10, and face scale 18/20. He showed mental distress from a relatively intense headache that was difficult to control, but had no evidence of psychiatric disorders such as depressive symptoms.

**Fig. 1:**
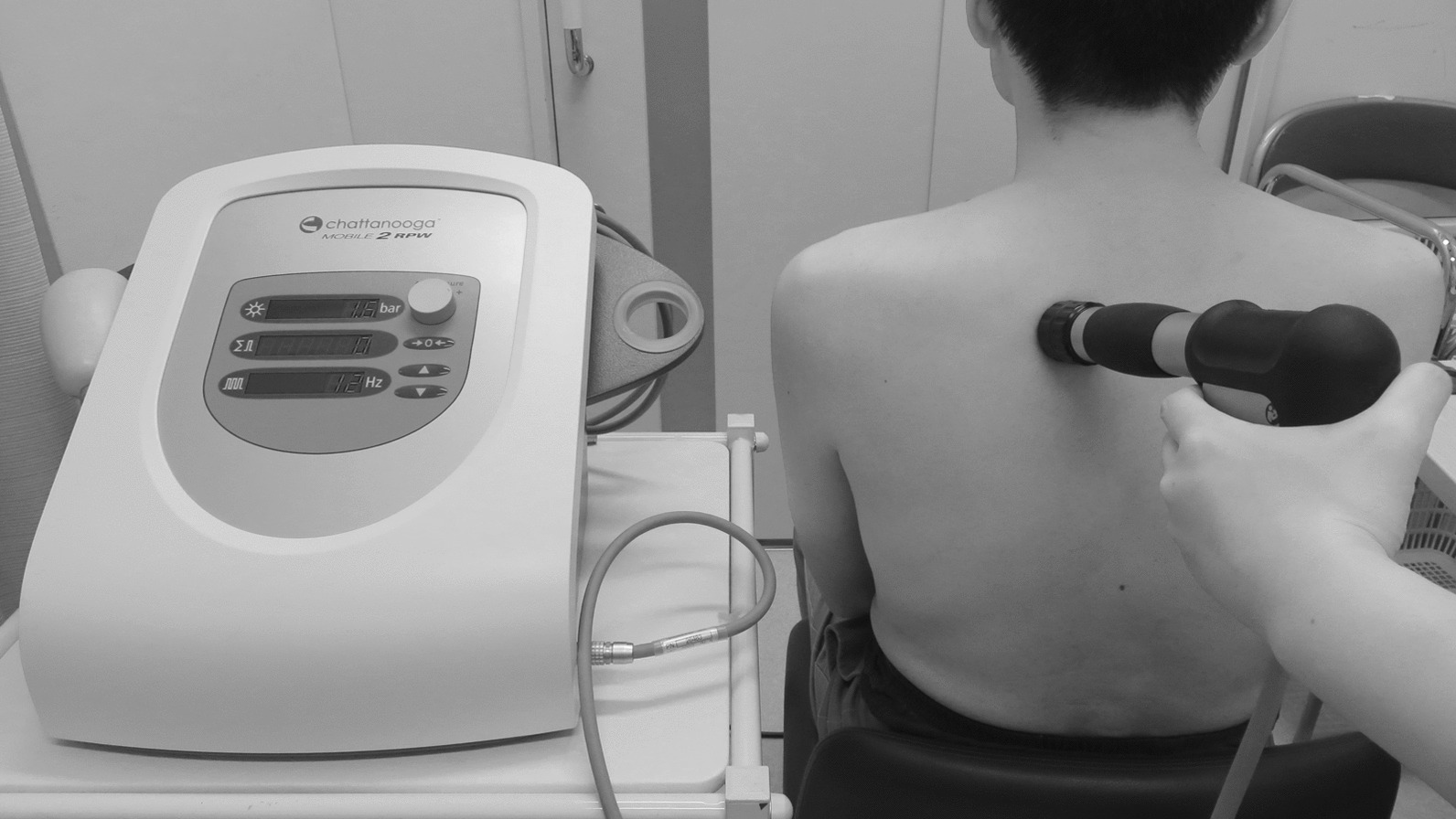
The extracorporeal shock wave therapy (ESWT) device we used on him and an image of the treatment. This instrument can emit radial pressure wave. This photograph is an image of ESWT; the man is not the patient in this case report

**Fig. 2:**
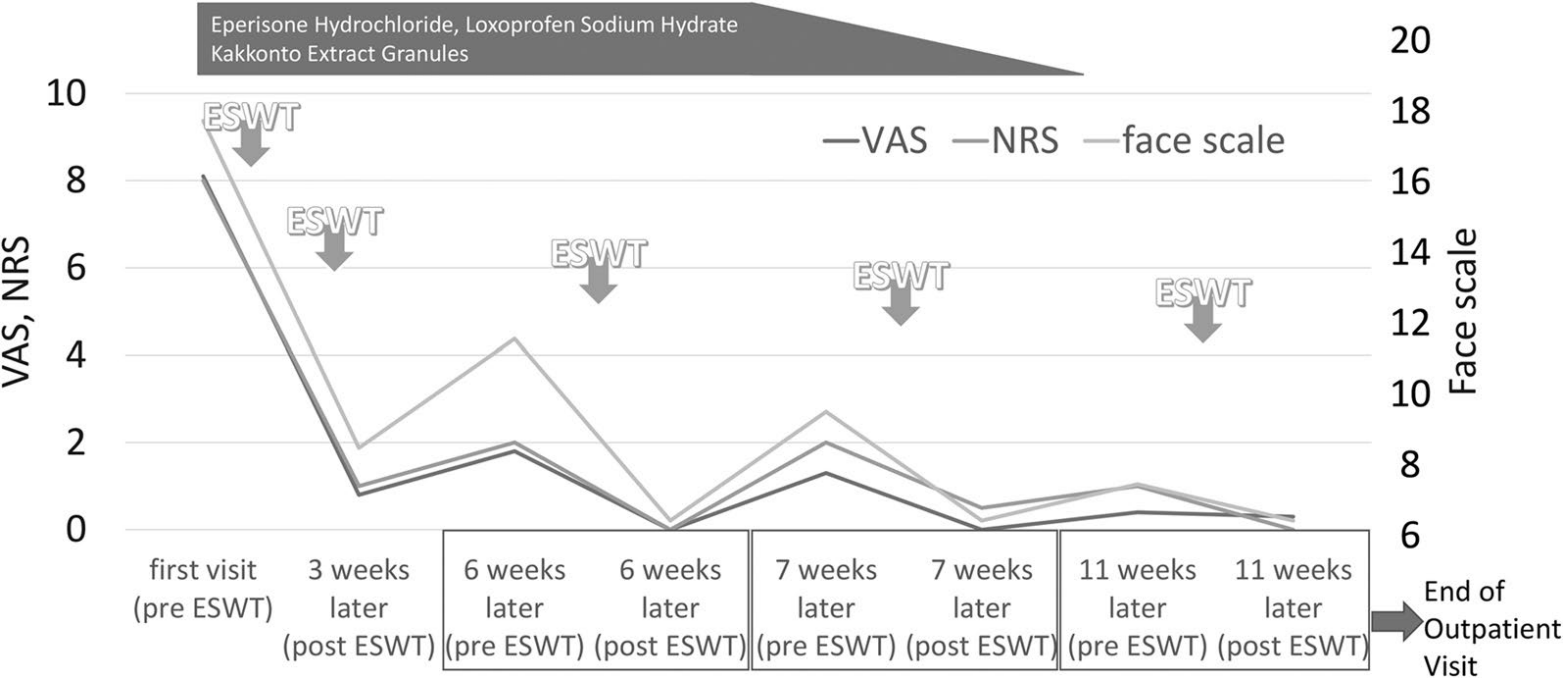
(From Ref [4], partially modified). Changes in the patient’s headache symptoms

Since BoNT-A is not covered by insurance for headache in Japan, at the initial visit he was treated with a total of 10 mL of 1% lidocaine intramuscularly injected into the bilateral splenius cervicis muscles, splenius capitis muscles, semispinalis capitis muscles, and trapezius muscles. He also received 2000 ESWT each into bilateral trapezius muscles at 1.4 bar and 12 Hz with the expectation of a massaging effect on the shoulder girdle. He agreed to undergo the treatment when he received them. ESWT (RPW) was performed using a pneumatic massager with a vibrating head (Stotz Medical AG, Switzerland; Intelect RPW Mobile; medical device approval number: 23000BZX00228000) (Fig. 1). Other than that, he had no problem regarding the use of health insurance coverage. In addition to eperisone hydrochloride and loxoprofen sodium hydrate, he was prescribed *Kakkonto* extract granules 2.5 g three times daily for 56 days, and benfotiamine 34.58 mg, pyridoxine hydrochloride 25 mg, and cyanocobalamin 250 µg (VITAMEDIN^®^ combination capsules B25) one capsule four times daily for 56 days, and was instructed to perform cervical muscle and shoulder girdle stretches and shoulder girdle exercises. At the T + 3 weeks outpatient visit, he underwent ESWT at 1.4 bar, 12 Hz for 2000 shots each to bilateral trapezius muscles, after which his headache improved to VAS 0.8/10, NRS 1/10, and face scale 9/20. He was continued on eperisone hydrochloride and loxoprofen sodium hydrate. At the T + 6 weeks outpatient visit, his headache was VAS 1.8/10, NRS 2/10, and face scale 12/20 before ESWT, slightly worse than that after ESWT at the T + 3 weeks outpatient visit, but significantly better than that at the initial visit. After ESWT of this visit, his headache improved further to the point that he felt no pain in the VAS (0/10) and NRS (0/10) assessment, and he reported his headache at 7/20 on the face scale. Because his headache was improved, the dosages of eperisone hydrochloride and loxoprofen sodium hydrate were reduced by self-adjustment. Because the immediate effect of ESWT wore off after a few days, he was administered ESWT one week later (T + 7 weeks). His headache was VAS 1.3/10, NRS 2/10, and face scale 10/20 before ESWT, which had worsened since the last ESWT, but overall his headache was gradually improving. After ESWT, his headache was lowered to VAS 0/10, NRS 0.5/10, and face scale 7/20, showing an immediate analgesic effect of ESWT. At T + 11 weeks, his headaches had improved, he had decreased his eperisone hydrochloride and loxoprofen sodium hydrate medications, and he was taking very few medications. His headache was VAS 0.4/10, NRS 1/10, and face scale 8/20 before ESWT, and VAS 0.3/10, NRS 0/10, and face scale 7/20 after ESWT. As his headache had improved to the point that he felt little pain even before ESWT, his outpatient visits to our hospital were terminated (Fig. 2). He did not develop any side effects associated with the treatment he received, including ESWT.

## Discussion

We performed multidisciplinary treatment including ESWT on a male patient (45 years old) with refractory CTTH. As a result, his headache symptoms improved markedly and generally disappeared. Routine chronic headache is a group of headache syndromes, mainly chronic migraine and CTTH [1, 5]. CTTH, the chronic form of TTH, is said to affect 0.5–4.8% of the population worldwide [6]. Although the present case was a middle-aged male, in general, TTH is more frequent in females than in males, and younger patients are more frequent than older patients [3].

His pain was evaluated using a VAS (rating on a scale of 0 to 10, with high scores reflecting more painful state) [7], the NRS (0 to 10 points; with high points reflecting more painful state) [8, 9], and the face scale, which is a sequence of 20 faces (0 to 20 points; with high points reflecting more painful state) [10]. The VAS scale measured the distance in 1 mm increments from one end to the point indicated by the patient on a line 10 cm long. For this reason, the data are shown to one decimal place. His headache, which was 18/20 on the face scale at the beginning of treatment, eventually decreased to 7/20 (Fig. 2). We surmised that 7/20 on the face scale that visually and intuitively expresses how he felt at that moment using a picture of a face was his normal status.

TTH is a common disease, and sleep dysregulation (lack of sleep or oversleeping) is often reported to trigger acute attacks [11]. However, the pathophysiology of headache is multifactorial and not fully understood [12]. In the present case, he had chronic headaches for about 20 years, and we speculated that the long hours spent working on a PC at home owing to the COVID-19 pandemic had increased intramuscular pressure in his shoulder girdle, which in turn had aggravated his headaches and made his symptoms worse. Although patients with CTTH who frequently or regularly use drugs are at risk of developing medication overuse headache (MOH) [13], we found no episodes of drug abuse in his history. We diagnosed CTTH because his symptoms were not pulsatile, but rather heavy-headed and tight, and they were persistent and long-lasting [14].

**Fig. 3:**
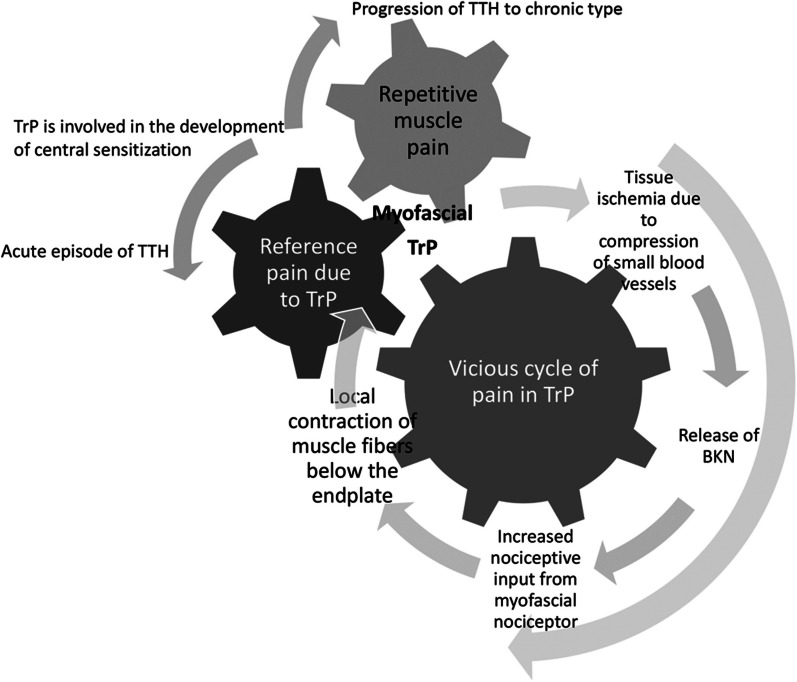
Vicious cycle of pain in tension-type headache (TTH). *TrP* trigger point

A myofascial TrP, of which multiple were found to be present in this case, is defined as a hyperirritable spot in skeletal muscle that is associated with a hypersensitive palpable nodule in a taut band [15]. The formation of myofascial TrPs is thought to be associated with muscle lesions that damage endplates and release excess acetylcholine (ACh) [16]. According to this hypothesis, reflex contractions of the muscle are triggered by nociceptive inputs from adjacent joints and muscles. A partial contraction of localized muscle fibers below the endplate occurs, and this contraction compresses small blood vessels, causing tissue ischemia. The pH may decrease with ischemia, and bradykinin is released [16]. Activation and sensitization of nociceptive nerve endings by the release of chemical mediators (bradykinin, serotonin, substance P) has then been proposed as the peripheral mechanism causing pericranial tenderness [17]. Indeed, a recent study states that higher levels of algogenic substances and lower pH levels are detected in active myofascial TrP compared with control tender points [17]. Thus, it can be said that a “vicious cycle of pain” occurs at myofascial TrP owing to the local ischemic state caused by muscle contraction, the release of chemical mediators such as bradykinin, and the induction of pain due to the stimulation of nociceptors (Fig. 3).

**Fig. 4:**
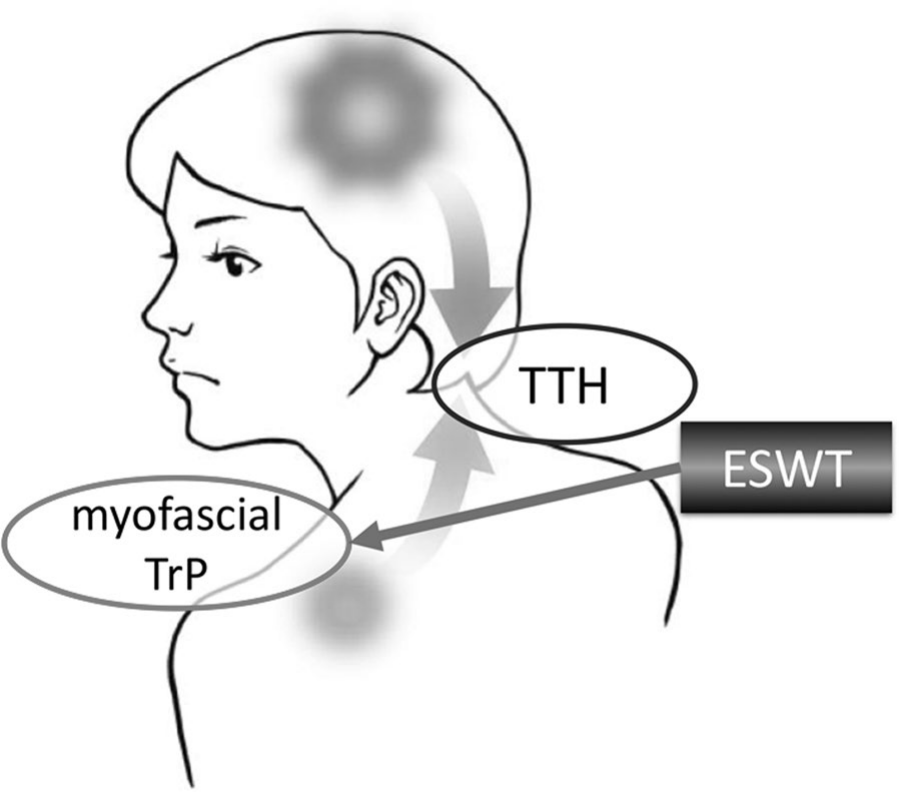
Myofascial trigger point (TrP) and tension-type headache (TTH). *ESWT* extracorporeal shock wave therapy. Illustration drawn by Mr. T. Yano of the Information Processing Office, Narita Hospital

The patient worked with a personal computer almost all day, every day, in a posture that placed continuous stress on the cervical spine, neck, and shoulder girdle muscles. Patients with refractory CDH often suffer from a vicious cycle of increasing pain [5]. With regard to the pathogenesis of CTTH, the role of central and peripheral mechanisms acting on symptom expression has been debated constantly [6]. The latest pain model of CTTH proposes that headache is at least partially a referred pain due to myofascial TrP in the posterior neck, head, and shoulder muscles, and that the TrPs would be the primary hyperalgesic zones responsible for the development of central sensitization in CTTH [17] (Fig. 4). In addition, a review has examined the peripheral factors involved in the mechanisms of TTH [18]. Here, our findings suggest that peripheral activation or sensitization of myofascial nociceptors may be most involved in the development of myalgia and acute episodes of TTH. In addition, recurrent episodes of myalgia may sensitize the central nervous system and cause TTH to progress to a chronic form, and muscle factors may also be involved in the chronicity of symptoms [18].

In the present case, multidisciplinary treatment, including ESWT, gradually relieved the headache, and most symptoms eventually disappeared. We honestly did not expect the treatment, including ESWT, to be so successful at the first visit; at the second visit (T + 3 weeks), we added an evaluation for pain because the patient had more symptom relief than we expected. As a result, headache was relieved before and after ESWT, among other things, suggesting a clear immediate effect on headache. ESWT is said to have many beneficial effects, including pain relief, angiogenesis, protein biosynthesis, cell proliferation, nerve and cartilage protection, and destruction of calcium deposits in musculoskeletal structures [19]. It has been shown that low-energy density extracorporeal shock wave (ESW) irradiation rapidly increases neuronal nitric oxide synthase (nNOS) activity and basal nitric oxide (NO) production [20, 21]. The increase in NO levels is then followed by inhibition of NF-kappaB activation [21]. Thus, ESW (a focused shock wave by electromagnetic method) irradiation is said to efficiently downregulate NF-kappaB activation and NF-kappaB-dependent gene expression (including induced NOS and TNF-α), suggesting a possible molecular mechanism for the anti-inflammatory effects of ESWT [20]. On the other hand, in a report [22] investigating the changes in neuromuscular transmission following ESW application, results suggest that ESW application to muscles leads to the degeneration of free nerve endings and induces a transient loss of nerve conduction function at the neuromuscular junction (NMJ). In addition to inhibiting excessive muscle contraction, ESWT may be effective in blocking the input of painful stimuli.

In order to break the “vicious cycle of pain,” we did not deploy the available forces (therapeutic means) in small doses, but rather, we tried to deploy greater forces from the initial stage of treatment. In other words, in addition to oral medications, including herbal medicines, injections of local anesthetics into the neck and shoulder girdle and ESWT, which is presumed to have an immediate effect on pain relief, were administered intensively in the early stages of treatment. In addition, we continued the ESWT in waves. Ibuprofen (400 mg) and aspirin (1000 mg) are recommended generally as first-line agents for the treatment of TTH [18], with other agents including baclofen, tizanidine, and botulinum toxin A [23]. The details of prescription choice in this case were unknown because the attending physician who prescribed the pain medication to him at that time is no longer with us and cannot be reached. In addition, several case reports [24, 25] have demonstrated the effectiveness of herbal medicines, such as *Kakkonto* extract granules, in improving headache. In our study, we prescribed *Kakkonto* extract granules as a herbal medicine. In fact, in this case, we injected 1% lidocaine, focusing on areas of perceived pain and areas of muscle stiffness in the neck and shoulder girdle. Although not an injectable drug, eperizone hydrochloride administered to patients with TTH has been shown to significantly reduce trapezius muscle stiffness and improved subjective headache symptoms [26]. As mentioned above, the reduction of muscle stiffness is thought to play an important role in relieving headache symptoms.

On the other hand, exercise therapy is generally effective for pain. In this case, the patient was instructed by a physician and a physical therapist in postures that do not put stress on the neck, as well as in independent training exercises to be performed between periods of desk work. We did not provide him with ongoing one-on-one therapy such as massage. Matsubara *et al.* [27] found that a light exercise program introduced as exercise therapy for not just headaches, but also for chronic pain patients who had difficulty with daily life and work, resulted in dramatic improvement of symptoms.

Considering the mechanism of TTH occurrence as described above and the antiinflammatory effects of ESWT and its influence on the neuromuscular unit (NMU), it is reasonable to conclude that ESWT acts mainly on the myofascial TrP, resulting in headache improvement, even if partial, especially in TTH where myofascial TrP is present, as in the present case. Therefore, ESWT should be considered as an important treatment option for TTH in the presence of myofascial TrP. In addition, to break the “vicious cycle of pain,” it is important to aggressively implement multimodal treatment, including herbal medicines, oral medications, and injections, from the initial stage of treatment.

The present study has several limitations. The combination of ESWT with other therapies may have had a positive effect on this patient’s condition, rather than ESWT alone. Though this multidisciplinary treatment may become a good treatment option for CTTH, this is a retrospective single case report, and the number of cases needs to be increased in the future.

## Conclusion

We report the case of a man (45 years old) with TTH who was treated successfully using a multimodal approach including ESWT in addition to standard treatment. For patients with TTH accompanied by myofascial TrP that is refractory to standard treatment, it is recommended to promptly consider aggressive multimodal treatment that includes ESWT.

## Data Availability

The datasets generated and/or analyzed during the current study are not publicly available, to protect personal information, but are available from the corresponding author on reasonable request.

## Abbreviations

BoNT-A: Botulinum toxin type A
CDH: Chronic daily headache
COVID-19: The coronavirus disease 2019
CT: Computed tomography
CTTH: Chronic tension-type headache
ESW: Extracorporeal shock wave
ESWT: Extracorporeal shock wave therapy
NO: Nitric oxide
NRS: Numerical rating scale
RPW: Radial pressure wave
TrP: Trigger point
TTH: Tension-type headache
VAS: Visual analog scale
